# Oesophageal cancer in women: tobacco, alcohol, nutritional and hormonal factors

**DOI:** 10.1054/bjoc.2001.1898

**Published:** 2001-08

**Authors:** S Gallus, C Bosetti, S Franceschi, F Levi, L Simonato, E Negri, C La Vecchia

**Affiliations:** Istituto di Ricerche Farmacologiche “Mario Negri”, Via Eritrea 62, Milan, 20157, Italy; International Agency for Research on Cancer, 150 Cours Albert Thomas, Lyon, Cedex 08, 69372, France; Centre di Riferìmento Oncologico Via Pedimontana, 33081 Aviano (PN), Italy; Registre vaudois des tumeurs, Institut universitaire de médecine sociale et preventive, CHUV-Falaises 1, Lausanne, 1011, Switzerland; Servizio di Epidemiologia dei Tumori, Registro dei Tumori del Veneto, Padua, Italy; Istituto di Statistica Medica e Biometria, Università degli Studi di Milano, Milan, Italy

**Keywords:** oesophageal cancer, female, alcohol drinking, smoking, diet, case–control studies

## Abstract

We analysed 3 case–control studies from Italy and Switzerland including 114 women with squamous cell oesophageal cancer and 425 controls. The multivariate odds ratio was 4.5 for heavy smoking and 5.4 for heavy alcohol drinking. Fruit intake, vegetable intake, oral contraceptive and HRT use were inversely related to oesophageal cancer. © 2001 Cancer Research Campaign http://www.bjcancer.com


British Journal of Cancer (2001) 85(3), 341–345
© 2001 Cancer Research Campaign
doi: 10.1054/ bjoc.2001.1898, available online at http://www.idealibrary.com on IDEAL®

http://www.bjcancer.com

Short Communication

### Oesophageal cancer in women: tobacco, alcohol, nutritional and hormonal factors

**S Gallus[1], C Bosetti[1], S Franceschi[2,3], F Levi[4], L Simonato[5], E Negri[1] and C La Vecchia[1,6]**

[1]Istituto di Ricerche Farmacologiche "Mario Negri", Via Eritrea 62, 20157 Milan, Italy; [2]International Agency for Research on Cancer, 150 Cours Albert Thomas, 69372 Lyon Cedex 08, France; [3]Centre di Riferìmento Oncologico, Via Pedimontana, 33081 Aviano (PN), Italy; [4]Registre vaudois des tumeurs, Institut universitaire de médecine sociale et preventive, CHUV-Falaises 1, 1011 Lausanne, Switzerland; [5]Servizio di Epidemiologia dei Tumori, Registro dei Tumori del Veneto, Padua, Italy; [6]Istituto di Statistica Medica e Biometria, Università degli Studi di Milano, Milan, Italy

**Summary** We analysed 3 case–control studies from Italy and Switzerland including 114 women with squamous cell oesophageal cancer and 425 controls. The multivariate odds ratio was 4.5 for heavy smoking and 5.4 for heavy alcohol drinking. Fruit intake, vegetable intake, oral contraceptive and HRT use were inversely related to oesophageal cancer. © 2001 Cancer Research Campaign http://www.bjcancer.com

**Keywords**: oesophageal cancer; female; alcohol drinking; smoking; diet; case–control studies

Squamous cell oesophageal cancer is relatively rare in women in developed countries, so, not surprisingly, most published data relate to men. In several countries, however, oesophageal cancer rates in women have tended to increase over the last few decades (Launoy et al, 1994; Negri et al, 1996; Desoubeaux et al, 1999; Levi et al, 1999). Among studies providing data on women, one from Italy (Tavani et al, 1993) and another from South America (Castellsagué et al, 1999), showed strong associations with alcohol and tobacco consumption. The relative risks for the highest level of alcohol consumption were around 2, and those for the highest level of tobacco consumption between 3 and 5 in both studies, apparently lower than those reported for men (Franceschi et al, 1990; Castellsagué et al, 1999). A case–control study from Shanghai, China, estimated that cigarette smoking accounted for 50% of oesophageal cancers in men, but only 14% in women (Gao et al, 1994). Among other factors investigated, hot mate drinking was strongly associated with oesophageal cancer in women in South America (Castellsagué et al, 2000), and a diet poor in fresh fruit or vegetables was directly related to risk in an Italian study (Tavani et al, 1993).

A role of female hormones has been suggested for several digestive tract cancers, including oral cavity (Bosetti et al, 2000a), stomach (La Vecchia et al, 1994) and colo-rectum (Fernandez et al, 2000). Little information, however, is available on any potential effect of female hormones on oesophageal carcinogenesis (IARC, 1999).

### SUBJECTS AND METHODS

The current analysis is based on data from 3 case–control studies of squamous cell oesophageal cancer, the first conducted in 1984–93 in the provinces of Milan and Pordenone (Fioretti et al, 1997), the second in 1992–97 in the provinces of Padua and Pordenone, and the greater Milan area, northern Italy (Franceschi et al, 2000), and the third, in 1992–99, in the Swiss Canton of Vaud (Levi et al, 2000). Data collected in Italy before 1991 have already been published (Tavani et al, 1993).

Cases considered in the present report were 114 women under 79 years (median age 63 years), with newly diagnosed histologically confirmed squamous cell oesophageal cancer, admitted to the major hospitals in the areas under study. Controls were 425 women (median age 62 years), admitted for acute, non-neoplastic conditions to the same hospitals. 40% of the controls were admitted for trauma, 21% for non-traumatic orthopaedic conditions, 24% for acute surgical disorders and 15% for miscellaneous other illnesses (including skin, eye, or ear disorders). Controls were frequency matched to cases by quinquennia of age and study centre, with a control-to-case ratio of 4, whenever possible.

Trained interviewers collected data using structured questionnaires, including information on socio-demographic characteristics, lifestyle habits, such as smoking and alcohol consumption, frequency of intake of selected food items, including major sources of carotene and retinol, menstrual, reproductive and hormonal factors, including lifelong use of oral contraceptives (OC) and hormone replacement therapy (HRT) during menopause. Reproducibility of the questionnaires was satisfactory (D'Avanzo et al, 1996, 1997). The questionnaire of the most recent study included more detailed and validated information about diet, in order to obtain estimates of the intake of several other nutrients (Franceschi et al, 1995; Decarli et al, 1996).

Cut-off points of the tertiles, based on discrete distributions of various foods and nutrients, were computed separately for the 3 studies. Odds ratios (ORs), and corresponding 95% confidence intervals (CI), were estimated using multiple logistic regression models (Breslow and Day, 1980) conditioned for age and study centre, and including terms for education, body mass index (BMI), non alcohol energy intake, tobacco and alcohol consumption.

*Received 16 March 2001*
*Revised 24 April 2001*
*Accepted 27 April 2001*

*Correspondence to: S Gallus*

**Table 1** Distribution of 114 female cases of oesophageal cancer and 425 controls according to age, education, and selected variables and corresponding odds ratios (OR) and 95% confidence intervals (CI), 1984–1999

| | Cases *n* (%) | Controls *n* (%) | OR[a] (95% CI) |
|---|---|---|---|
| Age | | | |
| <50 | 17 (14.9) | 60 (14.1) | |
| 50–54 | 12 (10.5) | 41 (9.7) | |
| 55–59 | 16 (14.0) | 62 (14.6) | |
| 60–64 | 28 (24.6) | 108 (25.4) | |
| 65–69 | 14 (12.3) | 59 (13.9) | |
| ≥70 | 27 (23.7) | 95 (22.4) | |
| Education (years) | | | |
| <7 | 66 (57.9) | 234 (55.1) | 1[b] |
| 7–11 | 33 (29.0) | 123 (28.9) | 0.49 (0.26–0.92) |
| ≥12 | 15 (13.2) | 68 (16.0) | 0.36 (0.16–0.80) |
| $\chi^2$ trend | | | 7.89 (*P* = 0.005) |
| Smoking habit | | | |
| Never smoker | 52 (45.6) | 295 (69.4) | 1[b] |
| Ex-smoker | 9 (7.9) | 45 (10.6) | 0.86 (0.35–2.09) |
| Current smoker (cigarettes/day) | | | |
| <15 | 16 (14.0) | 45 (10.6) | 1.74 (0.88–3.44) |
| 15–24 | 25 (21.9) | 32 (7.5) | 3.51 (1.76–7.01) |
| ≥25 | 12 (10.5) | 8 (1.9) | 4.54 (1.40–14.74) |
| $\chi^2$ trend[c] | | | 15.45 (*P* <0.001) |
| Alcohol intake (drinks/day) | | | |
| <1 | 29 (25.4) | 204 (48.0) | 1[b] |
| 1–2 | 50 (43.9) | 181 (42.6) | 1.99 (1.15–3.44) |
| ≥3 | 35 (30.7) | 40 (9.4) | 5.40 (2.70–10.80) |
| $\chi^2$ trend | | | 22.01 (*P* < 0.001) |
| Body mass index (kg/m$^2$)[d] | | | |
| <23 | 71 (62.3) | 145 (34.4) | 1[b] |
| 23–<26 | 20 (17.5) | 120 (28.5) | 0.25 (0.13–0.48) |
| ≥26 | 23 (20.2) | 156 (37.1) | 0.28 (0.15–0.51) |
| $\chi^2$ trend | | | 19.97 (*P* <0.001) |

[a]Estimates from multiple logistic regression models, conditioned for quinquennia of age and study centre, and including terms for education, body mass index, non-alcohol energy intake, tobacco and alcohol consumption. [b]Reference category. [c]Omitting ex-smokers. [d]The sum does not add up to the total because of some missing values.

### RESULTS

Table 1 shows the distribution of cases of squamous cell oesophageal cancer and controls according to age, education and selected characteristics. Cases were significantly less educated than controls (OR 0.36 for education ≥ 12 years compared to < 7 years), more frequently current smokers (OR 4.5 for ≥ 25 cigarettes per day), and heavy alcohol drinkers (OR 5.4 for ≥ 3 drinks per day). An inverse relation was observed with BMI (for the highest level, OR 0.28).

Table 2 gives the distribution of cases and controls by approximate tertiles of consumption of selected foods and micronutrients. Green vegetables, fresh fruit and carotene showed an inverse relation. For the highest tertile of intake compared to the lowest, the ORs were 0.45 for vegetables, 0.43 for fruit and 0.45 for carotene. There was no association with retinol intake.

Table 3 shows the distribution of cases and controls interviewed after 1992 by tertiles of olive oil and butter consumption and of several other nutrients. Most nutrients considered tended to be inversely related to the risk of oesophageal cancer, and the OR for the highest tertile of intake was 0.80 for lycopene, 0.85 for vitamin C, 0.59 for vitamin E, 0.11 for vitamin B6, 0.32 for folic acid, 0.34 for niacin and 0.57 for monounsatured fatty acids, though the trend in risk was significant only for vitamin B6, possibly because of the small numbers. Olive oil consumption showed a non-significant inverse relation (OR = 0.67), while butter consumption showed a positive but also non-significant relation (OR = 2.2).

The relation between oesophageal cancer risk and selected menstrual, reproductive and hormonal factors is shown in Table 4. The OR was higher for parous than for nulliparous women, but there was no significant trend in risk with parity. No association was observed with age at menarche, menopausal status and number of abortions. Cases reported earlier age at menopause (*P* for trend, 0.01). OC and HRT use were inversely associated to oesophageal cancer risk; for ever OC users the OR was 0.24, and for ever HRT users 0.32.

Table 5 shows the combined effect of alcohol intake and certain other factors. For the combined exposure of heavy drinking with heavy smoking the OR was 12.8, with low BMI was 10.1, with low vegetable intake was 8.9, and with low fruit intake was 13.9.

### DISCUSSION

This study, based on one of the largest datasets on squamous cell oesophageal cancer in women, confirms that in northern Italy and Switzerland tobacco smoking and alcohol drinking are the most

 *© 2001 Cancer Research Campaign*

**Table 2** Odds ratios (OR) and 95% confidence intervals (CI) of oesophageal cancer according to intake of green vegetables, fresh fruit, retinol, and carotene, 1984–1999

| | Tertiles, cases: controls, OR[a] (95% CI) | | | |
|---|---|---|---|---|
| | **1 (low)** | **2** | **3 (high)** | **$\chi^2$ trend (*P*)** |
| Green vegetables[b] | 41 : 84<br>1[c] | 21 : 90<br>0.61 (0.30–1.25) | 49 : 237<br>0.45 (0.24–0.85) | 5.93 (0.01) |
| Fresh fruit | 40 : 92<br>1[c] | 42 : 145<br>0.81 (0.44–1.51) | 32 : 188<br>0.43 (0.22–0.85) | 6.23 (0.01) |
| Retinol | 30 : 150<br>1[c] | 43 : 137<br>1.53 (0.84–2.78) | 41 : 138<br>1.40 (0.75–2.62) | 1.03 (0.31) |
| Carotene | 50 : 139<br>1[c] | 38 : 134<br>0.83 (0.45–1.53) | 26 : 152<br>0.45 (0.22–0.91) | 4.83 (0.03) |

[a]Estimates from multiple logistic regression models, conditioned by age and study centre, including terms for education, body mass index, non alcohol energy intake, tobacco and alcohol consumption. [b]The sum does not add up to the total because of some missing values. [c]Reference category.

**Table 3** Odds ratios (OR) and 95% confidence intervals (CI) of 47 female cases of oesophageal cancer and 157 controls, according to intake of selected nutrients, olive oil and butter, 1992–1999

| | Tertiles, cases: controls, OR[a] (95% CI) | | | |
|---|---|---|---|---|
| | **1 (low)** | **2** | **3 (high)** | **$\chi^2$ trend (*P*)** |
| Lycopene | 18 : 50<br>1[b] | 13 : 55<br>1.05 (0.35–3.10) | 16 : 52<br>0.80 (0.25–2.57) | 0.15 (0.70) |
| Vitamin C | 19 : 48<br>1[b] | 14 : 54<br>0.65 (0.23–1.84) | 14 : 55<br>0.85 (0.29–2.54) | 0.10 (0.75) |
| Vitamin E | 15 : 53<br>1[b] | 15 : 53<br>0.85 (0.26–2.71) | 17 : 51<br>0.59 (0.16–2.21) | 0.65 (0.42) |
| Vitamin B6 | 18 : 52<br>1[b] | 14 : 52<br>0.26 (0.07–0.95) | 15 : 53<br>0.11 (0.02–0.54) | 7.32 (< 0.01) |
| Folic acid | 17 : 50<br>1[b] | 12 : 56<br>0.43 (0.13–1.44) | 18:51<br>0.32 (0.07–1.43) | 2.10 (0.15) |
| Niacin | 17 : 50<br>1[b] | 14 : 56<br>0.34 (0.10–1.09) | 16 : 51<br>0.34 (0.10–1.16) | 2.94 (0.09) |
| Monounsaturated fatty acids | 14 : 53<br>1[b] | 15 : 53<br>0.47 (0.12–1.90) | 18 : 51<br>0.57 (0.12–2.81) | 0.20 (0.65) |
| Olive oil | 19 : 49<br>1[b] | 12 : 53<br>0.41 (0.13–1.26) | 16 : 55<br>0.67 (0.23–1.95) | 0.63 (0.43) |
| Butter | 10 : 58<br>1[b] | 17 : 54<br>1.34 (0.47–3.80) | 20 : 45<br>2.20 (0.73–6.65) | 1.93 (0.17) |

[a]Estimates from multiple logistic regression models, conditioned by age and study centre, and including terms for education, body mass index, non alcohol energy intake, tobacco and alcohol consumption. [b]Reference category.

important risk factors for this neoplasm in women as they are for men (Negri et al, 1992). In a study from northern Italy, the OR for men was 4.7 for heavy smokers and 6.0 for heavy drinkers (Franceschi et al, 1990). Although reported levels of alcohol consumption differ between men and women, our investigation indicates that, for highest levels of alcohol and tobacco consumption, the relative risks of oesophageal cancer are comparable in women and in men, as with smoking and lung and other tobacco-related neoplasms (Doll et al, 1980). This study confirms that women with oesophageal cancer tend to be less educated than controls (Tavani et al, 1993). Further, the present data indicate that cases have a lower BMI, though this could be partly a consequence of the cancer. Low BMI in the few years before diagnosis may be associated with decreased intake of protective nutrients. However, allowance for selected dietary indicators did not appreciably modify the association (Gallus et al, 2001). Our investigation also provides additional evidence that green vegetables, fresh fruit (Tavani et al, 1993; Castellsagué et al, 2000) and, consequently, carotene (Tavani et al, 1993) have a favourable effect on oesophageal cancer. The simultaneous exposure to alcohol and BMI, green vegetables or fruit seems to have an additive effect on oesophageal cancer (Breslow and Day, 1980). In contrast, simultaneous exposure to alcohol and tobacco has a multiplicative effect on oesophageal cancer, for women, as for men (Tuyns et al, 1977; Castellsagué et al, 1999). According to the multistage theory of carcinogenesis (Day and Brown, 1980), therefore, alcohol and tobacco would appear to act on different stages of the process, whereas alcohol and nutritional factors may act on the same

© 2001 Cancer Research Campaign 

**Table 4** Distribution of 114 women with oesophageal cancer and 425 controls according to gynecologic and hormonal factors, and corresponding odds ratio (OR) and 95% confidence interval (CI), 1984–1999

| | Cases | Controls | OR[a] (95% CI) |
|---|---|---|---|
| Age at menarche[b] | | | |
| <12 | 17 | 65 | 1[c] |
| 12–13 | 52 | 162 | 0.84 (0.40–1.73) |
| ≥14 | 44 | 198 | 0.60 (0.29–1.23) |
| $\chi^2$ trend | | | 2.51 ($P$ = 0.11) |
| Menopausal status[b] | | | |
| Pre + peri | 19 | 76 | 1[c] |
| Post | 93 | 349 | 1.78 (0.56–5.67) |
| Age at menopause[b,d] | | | |
| <45 | 29 | 67 | 1[c] |
| 45–49 | 27 | 80 | 0.75 (0.36–1.58) |
| ≥50 | 37 | 201 | 0.43 (0.22–0.83) |
| $\chi^2$ trend | | | 6.61 ($P$ = 0.01) |
| Number of births[b] | | | |
| 0 | 18 | 88 | 1[c] |
| 1 | 32 | 86 | 2.26 (1.03–4.96) |
| 2 | 39 | 133 | 2.22 (1.03–4.79) |
| ≥3 | 24 | 115 | 1.84 (0.82–4.16) |
| $\chi^2$ trend | | | 1.41 ($P$ = 0.24) |
| Number of abortions[b] | | | |
| 0 | 90 | 305 | 1[c] |
| ≥1 | 23 | 117 | 0.60 (0.33–1.07) |
| Oral contraceptive use | | | |
| Never | 110 | 392 | 1[c] |
| Ever | 4 | 33 | 0.24 (0.06–0.96) |
| Hormone replacement therapy use[d] | | | |
| Never | 89 | 308 | 1[c] |
| Ever | 4 | 41 | 0.32 (0.09–1.13) |

[a]Estimates from multiple logistic regression models, conditioned by age and study centre, and including terms for age (continuous term), education, body mass index, non alcohol energy intake, tobacco and alcohol consumption. [b]The sum does not add up to the total because of some missing values. [c]Reference category. [d]Post menopausal women only.

**Table 5** Odds ratios (OR) of oesophageal cancer in women and corresponding 95% confidence intervals (CI) according to the combined effect of alcohol drinking and smoking, BMI, vegetable and fruit intake, 1984–1999

| | Drinks/day, cases : controls, OR[a] (95% CI) | | |
|---|---|---|---|
| | < 1 drink | 1–2 drinks | ≥ 3 drinks |
| Smoking habit | | | |
| Never and ex smokers | 18 : 165<br>1[b] | 27:147<br>1.66 (0.85–3.25) | 16 : 28<br>5.79 (2.48–13.50) |
| Current smokers | 11 : 39<br>2.25 (0.95–5.33) | 23:34<br>5.52 (2.57–11.85) | 19 : 12<br>12.75 (5.09–31.96) |
| Body mass index[c] | | | |
| ≥25 kg/m$^2$ | 8 : 106<br>1[b] | 8 : 81<br>1.40 (0.49–4.03) | 9 : 15<br>7.82 (2.44–25.03) |
| <25 kg/m$^2$ | 21 : 95<br>3.06 (1.26–7.44) | 42 : 99<br>6.11 (2.62–14.26) | 26 : 25<br>10.12 (3.84–26.72) |
| Vegetable intake[c] | | | |
| High | 11 : 116<br>1[b] | 26 : 115<br>2.40 (1.08–5.33) | 17 : 23<br>7.50 (2.76–20.37) |
| Low | 17 : 76<br>2.35 (0.96–5.75) | 23 : 64<br>3.84 (1.59–9.26) | 17 : 17<br>8.94 (3.03–26.38) |
| Fruit intake | | | |
| High | 10 : 119<br>1[b] | 24 : 103<br>2.95 (1.28–6.79) | 10 : 17<br>5.74 (1.84–17.89) |
| Low | 19 : 85<br>2.92 (1.20–7.13) | 26 : 78<br>4.00 (1.68–9.55) | 25 : 23<br>13.89 (5.07–38.04) |

[a]Estimates from multiple logistic regression models, conditioned by age and study centre, and including terms for education, body mass index, non alcohol energy intake and tobacco consumption. [b]Reference category. [c]The sum does not add up to the total because of some missing values.

 *© 2001 Cancer Research Campaign*

stage(s). This again confirms the observations in males (Bosetti et al, 2000b; Franceschi et al, 2000), indicating that similar (dietary) patterns influence the risk of oesophageal cancer in women and men.

This study provides evidence of a potential favourable effect of female exogenous hormones (OC and HRT) (IARC, 1999), and suggests that age at menopause is inversely associated to squamous cell oesophageal cancer risk. This may be related to cigarette smoking, since smokers have earlier menopause (Baron et al, 1990). In contrast with oesophageal adenocarcinoma (Cheng et al, 2000), women with squamous cell oesophageal cancer were more frequently parous. This apparent association may be due to residual confounding by social class, although the social class correlates of oesophageal cancer seem to be changing (Bosetti et al, 2001).

In terms of population attributable risk (PAR) (Bruzzi et al, 1985; Mezzetti et al, 1996), tobacco smoking accounted for 31% of oesophageal cancer in women, alcohol drinking for 46%, and the combination of the 2 factors for 56%. The estimated PARs were 26% for low vegetable intake, 37% for low fruit, and 40% for vegetable and fruit combined. The PAR for the combination of alcohol, tobacco and vegetables was 75% (95% CI: 56–94%).

### ACKNOWLEDGEMENTS

The contributions of the Italian Association for Cancer Research, Milan, and the Italian (Milan), Swiss and Vaud Cancer Leagues are gratefully acknowledged. The authors thank Mrs J Baggott and MP Bonifacino for editorial assistance.

### REFERENCES

Baron JA, La Vecchia C and Levi F (1990) The antiestrogenic effect of cigarette smoking in women. *Am J Obstet Gynecol* **162**: 502–514

Bosetti C, Negri E, Franceschi S, Conti E, Levi F, Tomei F and La Vecchia C (2000a) Risk factors for oral and pharyngeal cancer in women: a study from Italy and Switzerland. *Br J Cancer* **82**: 204–207

Bosetti C, La Vecchia C, Talamini R, Simonato L, Zambon P, Negri E, Trichopoulos D, Lagiou P, Bardini R and Franceschi S (2000b) Food groups and risk of squamous cell esophageal cancer in Northern Italy. *Int J Cancer* **87**: 289–294

Bosetti C, Franceschi S, Negri E, Talamini R, Tomei F and La Vecchia C (2001) Changing socioeconomic correlates for cancers of the upper digestive tract. *Ann Oncol* **12**: 327–330

Breslow NE and Day NE (1980) *Statistical Methods in Cancer Research, Vol. 1. The Analysis of Case-Control Studies*. IARC Scientific Publication 32. IARC: Lyon, France

Bruzzi P, Green SB, Byar DP, Brinton LA and Schairer C (1985) Estimating the population attributable risk for multiple risk factors using case-control data. *Am J Epidemiol* **122**: 904–914

Castellsagué X, Muñoz N, De Stefani E, Victora CG, Castelletto R, Rolón PA and Quintana MJ (1999) Independent and joint effects of tobacco smoking and alcohol drinking on the risk of esophageal cancer in men and women. *Int J Cancer* **82**: 657–664

Castellsagué X, Muñoz N, De Stefani E, Victora CG, Castelletto R and Rolón PA (2000) Influence of mate drinking, hot beverages and diet on esophageal cancer risk in South America. *Int J Cancer* **88**: 658–664

Cheng KK, Sharp L, McKinney PA, Logan RFA, Chilvers CED, Cook-Mozaffari P, Ahmed A and Day NE (2000) A case-control study of oesophageal adenocarcinoma in women: a preventable disease. *Br J Cancer* **83**: 127–132

D'Avanzo B, La Vecchia C, Katsouyanni K, Negri E and Trichopoulos D (1996) Reliability of information on cigarette smoking and beverage consumption provided by hospital controls. *Epidemiology* **7**: 312–315

D'Avanzo B, La Vecchia C, Katsouyanni K, Negri E and Trichopoulos D (1997) An assessment, and reproducibility of food frequency data provided by hospital controls. *Eur J Cancer Prev* **6**: 288–293

Day and Brown (1980) Multistage models and primary prevention of cancer. *J Natl Cancer Inst* **64**: 977–989

Decarli A, Franceschi S, Ferraroni M, Gnagnarella P, Parpinel MT, La Vecchia C, Negri E, Salvini S, Falcini F and Giacosa A (1996) Validation of a food-frequency questionnaire to assess dietary intakes in cancer studies in Italy. Results for specific nutrients. *Ann Epidemiol* 1996; **6**: 110–118

Desoubeaux N, Le Prieur A, Launoy G, Maurel J, Lefevre H, Guillois JM and Gignoux M (1999) Recent time trends in cancer of the oesophagus and gastric cardia in the region of Calvados in France, 1978–1995: a population based study. *Eur J Cancer Prev* **8**: 479–486

Doll R, Gray R, Hafner B and Peto R (1980) Mortality in relation to smoking: 22 years' observations on female British doctors. *Br Med J* **280**: 967–971

Fernandez E, La Vecchia C, Balducci A, Chatenoud L, Franceschi S and Negri E (2000) Oral contraceptives and colorectal cancer risk: a meta-analysis. *Br J Cancer* **84**: 1–5

Fioretti F, Tavani A, La Vecchia C and Franceschi S (1997) Histamine-2-receptor antagonists and oesophageal cancer. *Eur J Cancer Prev* **6**: 143–146

Franceschi S, Talamini R, Barra S, Baron AE, Negri E, Bidoli E, Serraino D and La Vecchia C (1990) Smoking and drinking in relation to cancers of the oral cavity, pharynx, larynx, and esophagus in northern Italy. *Cancer Res* **50**: 6502–6507

Franceschi S, Barbone F, Negri E, Decarli A, Ferraroni M, Filiberti R, Giacosa A, Gnagnarella P, Nanni O, Salvini S and La Vecchia C (1995) Reproducibility of an Italian food frequency questionnaire for cancer studies. Results for specific nutrients. *Ann Epidemiol* **5**: 69–75

Franceschi S, Bidoli E, Negri E, Zambon P, Talamini R, Ruol A, Parpinel M, Levi F, Simonato L and La Vecchia C (2000) Role of macronutrients, vitamins and minerals in the aetiology of squamous-cell carcinoma of the oesophagus. *Int J Cancer* **86**: 626–631

Gao Y-T, McLaughlin JK, Blot WJ, Ji B-T, Benichou J, Dai Q and Fraumeni JF Jr (1994) Risk factors for esophageal cancer in Shanghai, China. I. Role of cigarette smoking and alcohol drinking. *Int J Cancer* **58**: 192–196

Gallus S, La Vecchia C, Levi F, Simonato L, Dal Maso L and Franceschi S (2001) Leanness and squamous cell oesophageal cancer. *Ann Oncol* 12: pages unavailable

IARC, International Agency for Research on Cancer (1999) *IARC Monograph on the Evaluation of Carcinogenic Risks to Humans, Vol. 72. Hormonal Contraception and Post-menopausal Hormonal Therapy*. IARC: Lyon

Launoy G, Faivre J, Pienkowski P, Milan C, Gignoux M and Pottier D (1994) Changing pattern of oesophageal cancer incidence in France *Int J Epidemiol* **23**: 246–251

La Vecchia C, D'Avanzo B, Franceschi S, Negri E, Parazzini F and Decarli A (1994) Menstrual and reproductive factors and gastric-cancer risk in women. *Int J Cancer* **59**: 761–764

Levi F, Lucchini F, Negri E, Boyle P and La Vecchia C (1999) Cancer mortality in Europe, 1990–1994, and an overview of trends from 1955 to 1994. *Eur J Cancer* **35**: 1477–1516

Levi F, Pasche C, Lucchini F, Bosetti C, Franceschi S, Monnier P and La Vecchia C (2000) Food groups and oesophageal cancer risk in Vaud, Switzerland. *Eur J Cancer Prev* **9**: 257–263

Mezzetti M, Ferraroni M, Decarli A, La Vecchia C and Benichou J (1996) Software for attributable risk and confidence interval estimation in case-control studies. *Comput Biomed Res* **29**: 63–75

Negri E, La Vecchia C, Franceschi S, Decarli A and Bruzzi P (1992) Attributable risks for oesophageal cancer in Northern Italy. *Eur J Cancer* **28A**: 1167–1171

Negri E, La Vecchia C, Levi F, Franceschi S, Serra-Majem L and Boyle P (1996) Comparative descriptive epidemiology of oral and oesophageal cancers in Europe. *Eur J Cancer Prev* **5**: 267–279

Tavani A, Negri E, Franceschi S and La Vecchia C (1993) Risk factors for esophageal cancer in women in Northern Italy. *Cancer* **72**: 2531–2536

Tuyns AJ, Péquignot G and Jensen OM (1977) Le cancer de l'oesophage en Ille-et-Vilaine en fonction des niveaux de consommation d'alcool et de tabac. Des risques qui se multiplient. *Bull Cancer* **64**: 45–60

© 2001 Cancer Research Campaign

